# Isolation and characterisation of 17 microsatellite DNA loci from RAD reduced-representation genomes for Asian warty newts, genus *Paramesotriton* (Caudata: Salamandridae)

**DOI:** 10.3897/BDJ.12.e113979

**Published:** 2024-02-02

**Authors:** Ming-Le Mao, Tao Luo, Wei Li, Ning Xiao, Huai-Qing Deng, Jiang Zhou

**Affiliations:** 1 School of Karst Science, Guizhou Normal University, Guiyang, China School of Karst Science, Guizhou Normal University Guiyang China; 2 School of Life Sciences, Guizhou Normal University, Guiyang, China School of Life Sciences, Guizhou Normal University Guiyang China; 3 Guiyang Healthcare Vocational University, Guiyang, China Guiyang Healthcare Vocational University Guiyang China

**Keywords:** microsatellite DNA loci, *Paramesotriton*, population genetics, conservation

## Abstract

Asian warty newts, genus *Paramesotriton*, are endemic to southern China and northern Vietnam. Despite the achievements in biodiversity, molecular systematics and biogeography of species in this genus, population genetic diversity studies are lacking due to the lack of economical and available genetic markers. In this study, we developed 17 highly polymorphic microsatellite loci from RAD simplified genomic data for the Asian warty newts, genus *Paramesotriton* and successfully completed cross-species amplification tests on 20 samples of four species of *Paramesotriton*. These microsatellite markers can be used as important tools to study population genetic structure, levels of gene flow, population differentiation, mating systems and landscape genetics within the genus *Paramesotriton* and, thus, to make scientific conservation decisions and actions for the conservation of these rare and endangered amphibians.

## Introduction

Asian warty newts, genus *Paramesotriton* (Caudata, Salamandridae), is a group of small-bodied, tailed amphibians found mainly in mountain streams throughout southern China and northern Vietnam, with 15 species currently recorded (Frost 2023). As an ancient amphibian, the genus *Paramesotriton* originated 25.42 million years ago and its species diversity history and current diversity pattern are driven by geology and paleoclimate (Luo et al. 2021, Luo et al. 2022) and has made some achievements in systematics and biogeography. Based on skeletal and morphological characters, Chinese Paramesotriton species were classified into three subgenera, Allomesotriton, Karstotriton and Paramesotriton (Fei and Ye 2016), a result that is also highly supported in phylogenetic analyses (Luo et al. 2022). However, little is still known about its genetic diversity. In fact, pollution of the habitat water environment, climate change and other adverse factors have put these species at different levels of endangerment, for example, the critically endangered *P.labiatus* and the endangered *P.yunwuensis*, *P.zhijinensis* and *P.guangxiensis* (IUCN 2023). Therefore, new genetic markers need to be developed to assess the genetic diversity of these species and used to understand their evolutionary genetic potential.

Microsatellite DNA markers are useful tools for understanding population genetic structure, mating systems, parentage analysis (Jones et al. 2010) and evaluating genetic resources and are widely used because of their high polymorphism, relatively small size and well-established analysis protocols (Vieira et al. 2016, Hu et al. 2021). With advances in next-generation sequencing technologies, it has become easier and faster to obtain microsatellite DNA loci from sequencing products for non-model organisms, such as transcriptomic and genomic data. Here, we first screened 164 microsatellite loci from reduced-representation genome data containing *P.zhijinensis*, *P.caudopunctatus*, *P.longliensis* and *P.maolanensis* and later described 17 highly polymorphic microsatellite DNA loci from them, which will help to study the genetic diversity of *Paramesotriton* species.

## Materials and Methods

Muscle tissue collected from the tails of individual individuals was used to extract genomic DNA using a genomic DNA extraction kit (NanoMagBio). PCR amplifications were performed in 10 µl reaction volume containing 1.0 µl of genomic DNA, 0.5 µl of each primer, 3.0 µl ddH_2_O and 5.0 µl 2 × Taq PCR Master Mix (GeneTech). The amplification conditions were as following: an initial denaturation at 95°C for 5 min; 10 cycles of denaturation at 95°C for 30 s, annealing at temperature 62°C–52°C for 30 s (use the touch-down procedure to drop 1°C per cycle) and extension at 72°C for 30 s; 25 cycles of denaturation at 95°C for 30 s, annealing at 52°C for 30 s and extension at 72°C for 30 s; and a final extension at 72°C for 20 min and final storage at 4°C. PCR products were separated on an ABI 3730XL Genetic Analyzer (Applied Biosystems), run through the simple sequence repeats sample analysis assay programme and analysed using GeneMarker 1.85 (Applied Biosystems).

Ultimately, 17 microsatellite loci were easily amplified and polymorphic and we proceeded to further amplification analysis of these loci. Allele size range, number of alleles (*Na*), effective number of alleles (*Ne*), observed heterozygosity (*Ho*), expected heterozygosity (*He*), Shannon’s Information Index (*I*) and genetic *fixation Index* (*I*) were analysed using GENETIX 4.0.5 (Belkhir 2004). Polymorphism information content (*PIC*) was calculated using Cervus 3.0.7 (Kalinowski et al. 2007).

## Results and Discussion

The 17 microsatellite DNA loci were examined using 20 tissue samples of four species of the genus *Paramesotriton*, *P.aurantius*, *P.zhijinensis*, *P.caudopunctatus* and *P.longliensis*. As shown in Table 1, all the remaining 14 were highly polymorphic (*PIC* > 0.5), except for microsatellite loci Cyn014, Cyn020 and Cyn024. The number of alleles (*Na*), effective number of alleles (*Ne*) and polymorphism information content (*PIC*) ranged from 3–12, 1.473–8.579 and 0.295–0.874, observed heterozygosity (*Ho*) ranged from 0.00–0.75, expected heterozygosity (*He*) ranged from 0.321–0.883 and the Shannon's Information Index ranged from 0.601–2.330 (Table 1). Except for the microsatellite locus Cyn023, the remaining 16 loci deviated from Hardy-Weinberg theory (*F* > 0), indicating that there might be many homozygotes in the population of *Paramesotriton*. This deviation from Hardy-Weinberg may be due to one or more factors, including insufficient sample size, substructure of the sample, the presence of zero alleles or inbreeding (Wang et al. 2010), as most of these salamanders were found to have only one geographic population and may not be representative of a true random mating population. Our cross-species amplification experiments on a total of 20 samples from seven populations of these four species showed that all 17 microsatellite primers were able to amplify all species, with only a few population samples failing to amplify successfully (Table 1).

Most salamanders have larger genomes, which presents difficulties in conducting genetic studies of salamander taxa, for example, *Ambystomamexicanum* has a genome of ~ 32 G (Nowoshilow et al. 2018). Although population genetic data and microsatellite markers have been previously contributed for Chinese-distributed tailed amphibians, such as the Chinese giant salamander (Yan et al. 2018) and the Shangcheng stout salamander (Wang et al. 2010). For Asian warty newts, genus *Paramesotriton*, there is still a lack of economical and practical population genetic markers, which hinders our understanding of genetic diversity and limits conservation actions. The microsatellite markers successfully amplified cross-species in this study and provided enough variation to study the population structure, gene flow levels, mating systems and landscape genetics of species within *Paramesotriton*, providing scientific genetic data for further assessment of the endangerment level of *Paramesotriton* and for conservation actions.

## Figures and Tables

**Table 1.** Characterisation of 17 microsatellites for species of the genus *Paramesotriton*. Abbreviations: N = Number of samples successfully amplified cross-species; *Na* = Number of alleles; *Ne* = No. of Effective Alleles; *I* = Shannon’s Information Index; *Ho* = Observed heterozygosity; *He* = Expected heterozygosity; *F* = Fixation Index; *PIC* = polymorphism information content.

Locus	Primer sequence (5'−3') (F, forward; R, reverse)	Repeatmotif	Size range (bp)	*N*	*Na*	*Ne*	*I*	*Ho*	*He*	*F*	*PIC*
Cyn013	F: TCCCTGTGTCGGTTCTTCTC R: CCAGGGAAGCCGGTATTGAT	(AG)_7_	173–181	16	3	2.462	0.974	0	0.594	1.000	0.511
Cyn014	F: AGGCTTGAAGACTTGGCTCT R: GCTGTCGCTCTAACTAGGCT	(GA)_6_	211–214	19	3	1.551	0.660	0	0.355	1.000	0.328
Cyn015	F: GTGAGCAGTGTGTGTGTATGT R: ACTTTCCGACTCCAACCA	(TG)_7_	191–198	18	4	3.878	1.371	0.167	0.742	0.775	0.694
Cyn020	F: TTCGAATTAGGGGAGCTGGG R: GGGCAAAGAAAGCAGGTTCA	(AT)_7_	216–221	19	3	1.473	0.601	0.125	0.321	0.611	0.295
Cyn023	F: TCCACATCTTCCTTTCGATAGC R: CTGTGAAATGGACTGGT	(CT_)8_	220–234	20	7	3.704	1.528	0.700	0.730	0.041	0.688
Cyn024	F: GTGCTCTCCTTGTTTGGGTG R: GCCTGCTGTGCTATTGTCAG	(AT)_6_	241–243	19	3	1.853	0.776	0.125	0.460	0.728	0.397
Cyn047	F: ACCATTATGACTAAACCCAGCA R: AAGATAAGAGCGGACCGGAG	(AT)_7_	210–217	20	5	2.721	1.180	0.150	0.633	0.763	0.578
Cyn053	F: GCGTAGATGTAATGAAAGCAGGA R: GCTCTCTCACTTTCCCCAGT	(TC)_9_	193–219	19	7	5.508	1.833	0.474	0.818	0.421	0.797
Cyn055	F: CCTGTGCCAGTGTGAATTGT R: ATGTACATGCCCCACCAGAA	(TG)_9_	159–186	17	12	8.579	2.330	0.750	0.883	0.151	0.874
Cyn062	F: GGGATTCGGTAAAAGCAGCC R: CATGAGCAGCCCACAGAAA	(CTTA)_5_	172–187	19	3	1.551	0.660	0.211	0.355	0.408	0.328
Cyn063	F: TCAGACACAATGATGCCAAACA R: CAGTGCCCAGATACCCCTAG	(ATCA)_7_	118–134	20	5	2.807	1.298	0.400	0.644	0.379	0.609
Cyn084	F: CTTTTCCATGCCTGTCCACA R: CCCAGGTGTGAGTGTGCTAT	(CATT)_7_	183–208	20	10	5.517	1.931	0.500	0.819	0.389	0.797
Cyn085	F: TCCTGTGACTTAGTTTTGGCAC R:TGAAGACAGACACAGACAATGA	(AG)_7_	236–245	20	5	2.963	1.328	0.250	0.662	0.623	0.626
Cyn086	F: TGAGGAGAGGAGAGGGAACA R: CCGCTGTCTCTCTCCATCTT	(AG)_6_	159–167	20	5	2.614	1.242	0.150	0.618	0.757	0.582
Cyn119	F: GCTGAACTTGCATGTCATAGAA R: GTTGGCCATCTGTAGTGCT	(AAT)_6_	150–159	20	5	2.360	1.097	0.300	0.576	0.479	0.528
Cyn120	F: AACGTCCCTGAAACCTTTGT R: GCTTTACACCTGCCACATGT	(AT)_6_	202–205	18	4	3.096	1.186	0.500	0.677	0.261	0.610
Cyn140	F: AGATGTGGGAGGTCATTTGGA R: AATGAGGTAAAGTCCCGGGG	(CA)_1_	145–155	19	6	4.276	1.582	0.211	0.766	0.725	0.730

## References

[B10532270] Belkhir K (2004). logiciel sous Windows TM pour la génétique des populations. http://www.genetix.univ-montp2.fr/genetix/genetix.

[B10532278] Fei L, Ye C. Y. (2016). Amphibians of China.

[B10532286] Frost D. R Amphibian species of the world: An online reference. https://amphibiansoftheworld.amnh.org.

[B10532294] Hu Y, Fan H, Chen Y, Chang J (2021). Spatial patterns and conservation of genetic and phylogenetic diversity of wildlife in China. Science Advances.

[B10532322] IUCN The IUCN Red List of Threatened Species. https://www.iucnredlist.org.

[B10532332] Jones A. G, Small C. M, Paczolt K. A, Ratterman N. L (2010). A practical guide to methods of parentage analysis. Molecular Ecology Resources.

[B10532341] Kalinowski S. T, Taper M. L, Marshall T. C (2007). Revising how the computer program CERVUS accommodates genotyping error increases success in paternity assignment. Molecular Ecology.

[B10532350] Luo T, Wen H, Gao K, Zhou J (2021). Phylogeography and cryptic species diversity of *Paramesotritoncaudopunctatus* species group (Salamandridae: Paramesotriton) in Guizhou, China. Asian Herpetological Research.

[B10532359] Luo T, Yan S. S, Xiao N, Zhou J. J (2022). Phylogenetic analysis of combined mitochondrial genome and 32 nuclear genes provides key insights into molecular systematics and historical biogeography of Asian warty newts of the genus *Paramesotriton* (Caudata: Salamandridae). Zoological Research.

[B10532368] Nowoshilow S, Schloissnig S, Fei J. F, Dahl A (2018). The axolotl genome and the evolution of key tissue formation regulators. Nature.

[B10532377] Vieira M. L. C, Santini L, Diniz A. L, Munhoz C. D.F (2016). Microsatellite markers: what they mean and why they are so useful. Genetics and Molecular Biology.

[B10532386] Wang H, Shi W, Zhu L, Luo X (2010). Isolation and characterization of fourteen polymorphic tetranucleotide microsatellites for Shangcheng stout salamander (*Pachyhynobiusshangchengensis*). Conservation Genetics Resources.

[B10532397] Yan F, Lü J, Zhang B, Yuan Z (2018). The Chinese giant salamander exemplifies the hidden extinction of cryptic species. Current Biology.

